# On the Treatment of Young Permanent Teeth, That Require Plugging

**Published:** 1847-12

**Authors:** 


*On the treatment of Young Permanent Teeth, that require Plugging.* By J. F. B. Flagg, M. D.

In the course of a series of microscopic observations upon the human teeth, in which I have been engaged for more than two years past, I was early led to observe the striking difference of density in the bony portions of teeth of different ages ; particularly in their more external parts, as the bone approaches either toward the enamel or cortex.

In two preparations, consisting of transverse sections at the neck, one, an adult tooth, the other of the age of eight years, I have observed this peculiar development most decidedly illustrated. A section of the older tooth being prepared sufficiently to allow the light to pass between its pores, when subjected to a powerful lens, presented a uniformly fibrous, or striated appearance, from the edge bordering upon the chamber of the nervous pulp, quite to its outer surface, with the slight exception of the *cortex* at this part of the tooth ; that being beautifully defined by its clustering stars.

A similar section of the young permanent molar, although it appeared equally opaque as the other at its *inner* portion, yet it gradually became less so, for about two-thirds of the way towards its outer circumference, when all its fibrous appearance was lost, and the external third became perfectly translucent.

Much difficulty has been experienced in the practice even of our best operators in regard to saving these early permanent teeth. It is not unfrequently necessary to fill the same cavity twice or three times, at periods varying from one to three years, before we can we can confidently pronounce it to be an operation *lasting and permanently useful.* The probable reason for this is the change which is constantly progressing within the tooth, necessary to its growth and full development ; the capillary tubes, in the immediate vicinity of the cavity so filled, evidently acting to its detriment. I think it reasonable to suppose that this condition may be superinduced by vital action being in contact with a foreign substance. Be this as it may, having pursued the following practice with uniform success, I recommend it to others desirous of benefitting the condition of young sufferers in this respect.

After removing every particle of decay, I devote as much time to *burnishing* the bony surface as is necessary to close the mouths of the tubuli opening into the cavity. This should be done with a smooth instrument, capable of reaching every portion of exposed bone, and with sufficient strength to cause the bone to present under the instrument somewhat the feeling of *enamel;* then wipe dry, and fill *full, solid,* and *finish.—Med, Examiner.*

